# Hepatic Artery Pseudoaneurysm After Laparoscopic Cholecystectomy: A Rare Cause of Gastrointestinal Bleeding

**DOI:** 10.7759/cureus.99431

**Published:** 2025-12-17

**Authors:** Malyka Batool, Alaita Fatima Bakhtiari, Smavia Hameed, Abdullah Saeed, Imran Ali Syed, Usman Iqbal Aujla

**Affiliations:** 1 Gastroenterology and Hepatology, Pakistan Kidney and Liver Institute and Research Centre, Lahore, PAK; 2 Radiology, Pakistan Kidney and Liver Institute and Research Centre, Lahore, PAK

**Keywords:** acute pancreatitis, endovascular stenting, gastrointestinal bleeding, hemobilia, hepatic artery pseudoaneurysm, laparoscopic cholecystectomy

## Abstract

Hepatic artery pseudoaneurysm (HAP) is a rare but serious complication after hepatobiliary surgery or pancreatitis. We present the case of a 33-year-old man with a history of recurrent pancreatitis complicated by portal vein thrombosis and gastric varices. Extensive workup did not reveal any etiology for his recurrent pancreatitis other than gallbladder sludge and stones. Therefore, he underwent a laparoscopic cholecystectomy as a potential source of pancreatitis. Post-cholecystectomy, he remained asymptomatic for a couple of months and later had recurrent episodes of upper gastrointestinal (GI) bleeding. He was investigated at the local hospital for his recurrent bleeding episodes and was advised to get injection sclerotherapy for his gastric varices. He presented to our institute following a massive fresh bleeding per rectum and severe anemia (hemoglobin of 3.3 g/dL). After initial resuscitation, an endoscopy was performed, which revealed well-covered gastric varices without any red warning sign or stigma of recent bleeding. There was a large yellow-based ulcer with surrounding edema in the duodenal bulb with visible pulsations; however, no vessels were visible. Subsequently, a CT angiogram was performed, which showed a large right HAP. He was immediately transferred to the interventional radiology suite, and endovascular stenting was successfully performed. The patient remained under observation for 48 hours without any further drop in hemoglobin or GI bleeding episodes. He was later discharged with outpatient follow-up. Although uncommon, a pseudoaneurysm after laparoscopic cholecystectomy should be suspected in patients with unexplained GI bleeding and a relevant surgical history. Imaging plays a key role in diagnosis, and endovascular management, such as stenting or embolization, is often preferred. This case highlights the importance of maintaining a high index of suspicion and acting quickly to prevent life-threatening complications.

## Introduction

Hepatic artery pseudoaneurysm (HAP) is an uncommon but potentially life-threatening vascular complication characterized by disruption of the arterial wall, leading to blood accumulation in surrounding tissues. HAPs have been reported in relation to acute cholecystitis, xanthogranulomatous cholecystitis, acute pancreatitis, trauma, liver transplantation, and percutaneous interventions [1,2]. The incidence of all hepatic artery aneurysms is estimated at approximately 0.002%, and approximately 50% of hepatic artery aneurysms are pseudoaneurysms [3]. HAP is considered a rare complication in patients undergoing surgical treatment for liver and gallbladder diseases through laparoscopic surgery [4]. The incidence of pseudoaneurysm after laparoscopic surgery ranges from 0.06% to 0.6% [5]. The most commonly involved artery is the splenic artery, followed by the gastroduodenal artery, while the hepatic artery is among the least common [6]. HAPs have been successfully treated using a variety of interventional methods, including endovascular embolization, coil embolization, and arterial stent grafting [7]. Here, we present the case of a young gentleman who developed recurrent upper gastrointestinal (GI) bleeding following laparoscopic cholecystectomy and a history of acute pancreatitis.

## Case presentation

A 33-year-old man presented to the emergency department with hypotension and fresh bleeding per rectum for one day. It was associated with generalized abdominal pain and non-bloody vomiting. His past medical history was significant for recurrent episodes of acute pancreatitis over the last seven years. It was complicated by the development of portal vein thrombosis and resultant left-sided portal hypertension. The raised portal pressure resulted in the formation of gastric fundal varices, diagnosed early during the course of his illness when he had an episode of hematemesis, which was managed endoscopically by injection sclerotherapy at that time. His bleeding remained well controlled for many years; however, he reported several episodes of acute pancreatitis in the meantime. Evaluation for the underlying etiology of recurrent pancreatitis identified gallbladder sludge as a potential source. Hence, it was decided to remove his gallbladder, and a laparoscopic cholecystectomy was performed approximately eight months before presentation at our institute.

Post-cholecystectomy, he remained asymptomatic for a couple of months and then started experiencing recurrent episodes of hematemesis and hematochezia. He described these episodes as abrupt and massive, which often left him hypotensive. On multiple occasions, these episodes occurred while driving, necessitating urgent transfer to the nearest hospital for stabilization. Despite undergoing endoscopic evaluation at an external facility, a clear etiology for the bleeding was not identified, and it was assumed that the gastric varix was the potential cause of his bleeding. Injection sclerotherapy was recommended, but the patient failed to follow up and eventually presented to us in a critical condition.

At presentation, he was hypotensive and severely anemic, with a hemoglobin level of 3.3 g/dL. After initial resuscitation with intravenous fluids and blood transfusions, he was transferred to the intensive care unit for close monitoring and escalation of care.

Laboratory investigations revealed normal liver function tests (total bilirubin = 1.55 mg/dL, aspartate aminotransferase = 27 IU/L, alanine aminotransferase = 26 IU/L, alkaline phosphatase = 129 IU/L, and serum albumin = 3.17 g/dL). Coagulation profile and renal function tests were within normal limits. Tumor markers showed a serum alpha-fetoprotein level of 1.99 ng/mL (Table 1).

**Table 1:** Laboratory investigations.

Parameter	Patient’s value	Reference range
Serum albumin	3.17 g/dL	3.5–5 g/dL
Alpha-fetoprotein	1.99 ng/mL	≤7.0 ng/mL
Hemoglobin	3.3 g/dL	12.3–16.6 g/dL
White cell count	6.4 × 10³/μL	4.6–11.38 × 10³/μL
Platelets	100 × 10⁹/L	150–450 × 10⁹/L
International normalized ratio	1.23	0.7–1.5
Serum creatinine	0.79 mg/dL	0.7–1.2 mg/dL
Serum sodium	137 mEq/L	135–145 mEq/L
Serum potassium	4.3 mEq/L	3.5–5.1 mEq/L
Total bilirubin	1.55 mg/dL	0.2–1.2 mg/dL
Alanine aminotransferase	27 IU/L	0–55 IU/L
Aspartate aminotransferase	26 IU/L	5–34 IU/L
Alanine aminotransferase	129 IU/L	40–130 IU/L

Following initial resuscitation, the patient’s hemoglobin levels improved and remained stable before endoscopic evaluation (Table 2).

**Table 2:** Serial trends of complete blood count from admission to follow-up.

Complete blood count	On presentation	On the 2nd day of admission	On discharge	On the 6-month follow-up	Reference range
Hemoglobin	3.3 g/dL	5.1 g/dL	7.1 g/dL	9.7 g/dL	12.3–16.6 g/dL
Total leukocyte count	6.4 × 10³/μL	9.04 × 10³/μL	8.6 × 10³/μL	7.2 × 10³/μL	4.6–11.38 × 10³/μL
Platelets	100 × 10⁹/L	92 × 10⁹/L	82 × 10⁹/L	139 × 10⁹/L	150–450 × 10⁹/L

**Figure 1:**
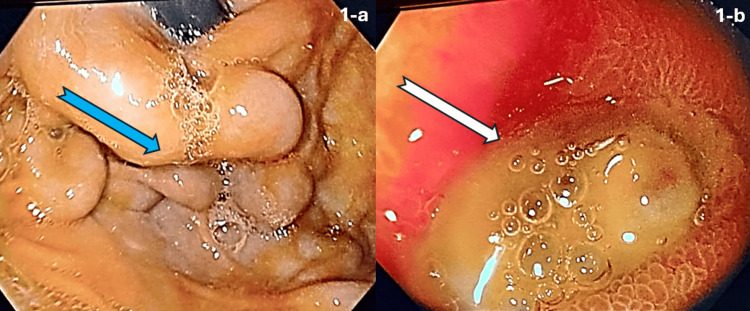
Endoscopic images. (a) Endoscopic image of the gastric fundus demonstrating isolated fundal varices (blue arrow) with a smooth, glistening surface and no stigmata of recent bleeding. (b) Endoscopic image of the first part of the duodenum showing a 1.5 cm, yellow-based anterior wall ulcer (white arrow) with surrounding erythema and edema, without visible vessels, clots, or active bleeding.

After achieving a hemoglobin level of 7 g/dL, an endoscopy was performed. It showed three columns of well-covered (grade I) esophageal varices [8]. A careful examination of the stomach revealed a medium-sized isolated bunch of fundal varices. The surface of the gastric varix appeared smooth and glistening without any red signs or stigmata of recent or active bleeding (Figure 1a). However, the first part of the duodenum revealed a large yellow-based ulcer measuring up to 1.5 cm located on the anterior wall. There was marked erythema and edema of the surrounding mucosa; however, no visible vessels, clots, or stigmata of active bleeding were observed (Figure 1b).

**Figure 2:**
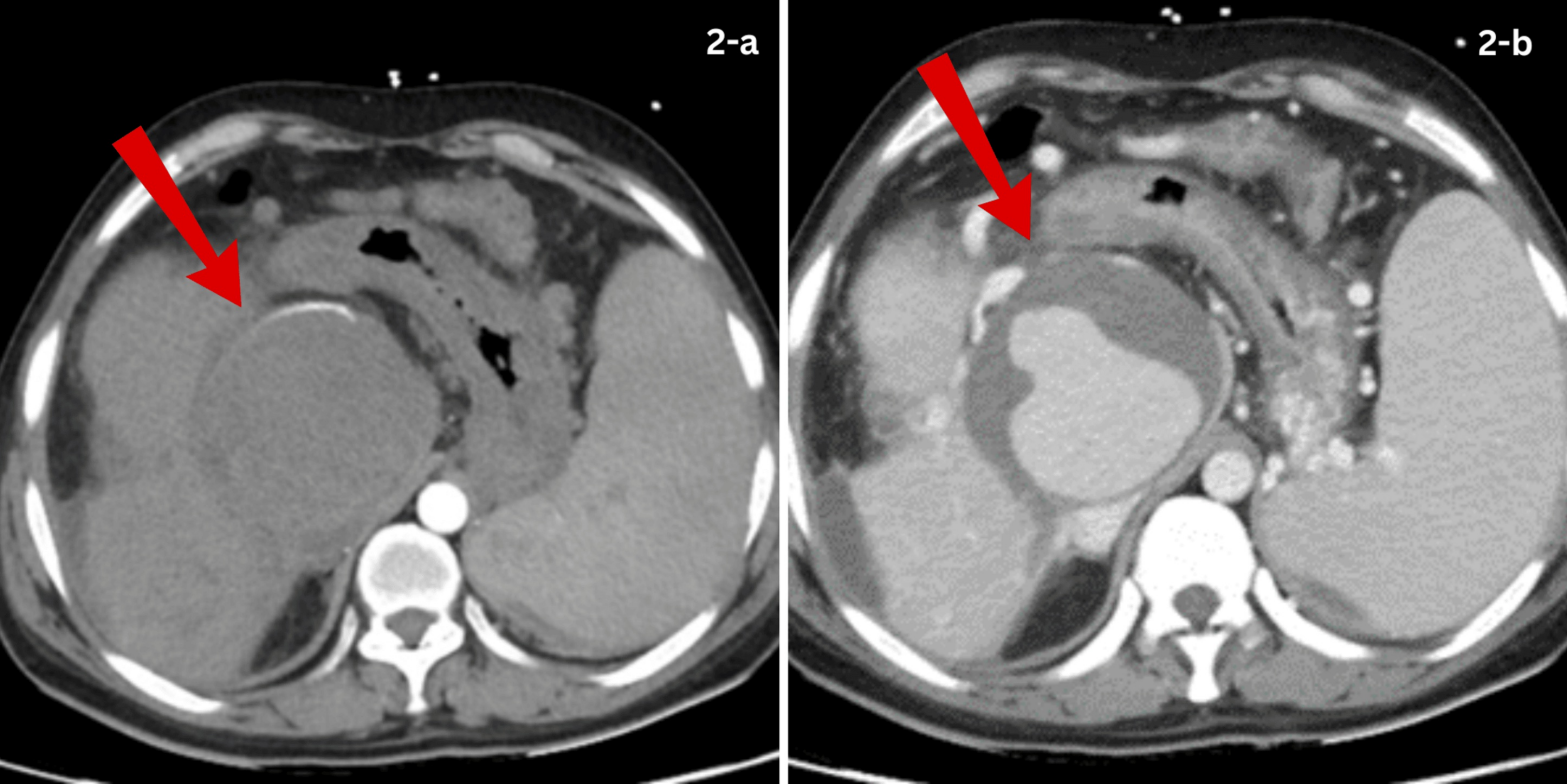
CT images of the hepatic artery pseudoaneurysm. (a) A large right hepatic artery pseudoaneurysm (7.3 x 6.8 cm) displacing the hepatic artery and demonstrating flow in the pseudoaneurysm. (b) Portal venous phase image of the hepatic artery pseudoaneurysm with partial thrombosis of the pseudoaneurysm.

A CT triphasic scan was performed to rule out the cause of bleeding. The CT imaging demonstrated a large peripherally thrombosed pseudoaneurysm in the subhepatic region arising from the hepatic artery, measuring approximately 7.3 × 6.8 cm, associated with cavernous transformation of the portal vein, mild intrahepatic biliary dilatation, suggestive of portal biliopathy, subhepatic fluid collections, and features of chronic pancreatitis (Figures 2a, 2b).

**Figure 3:**
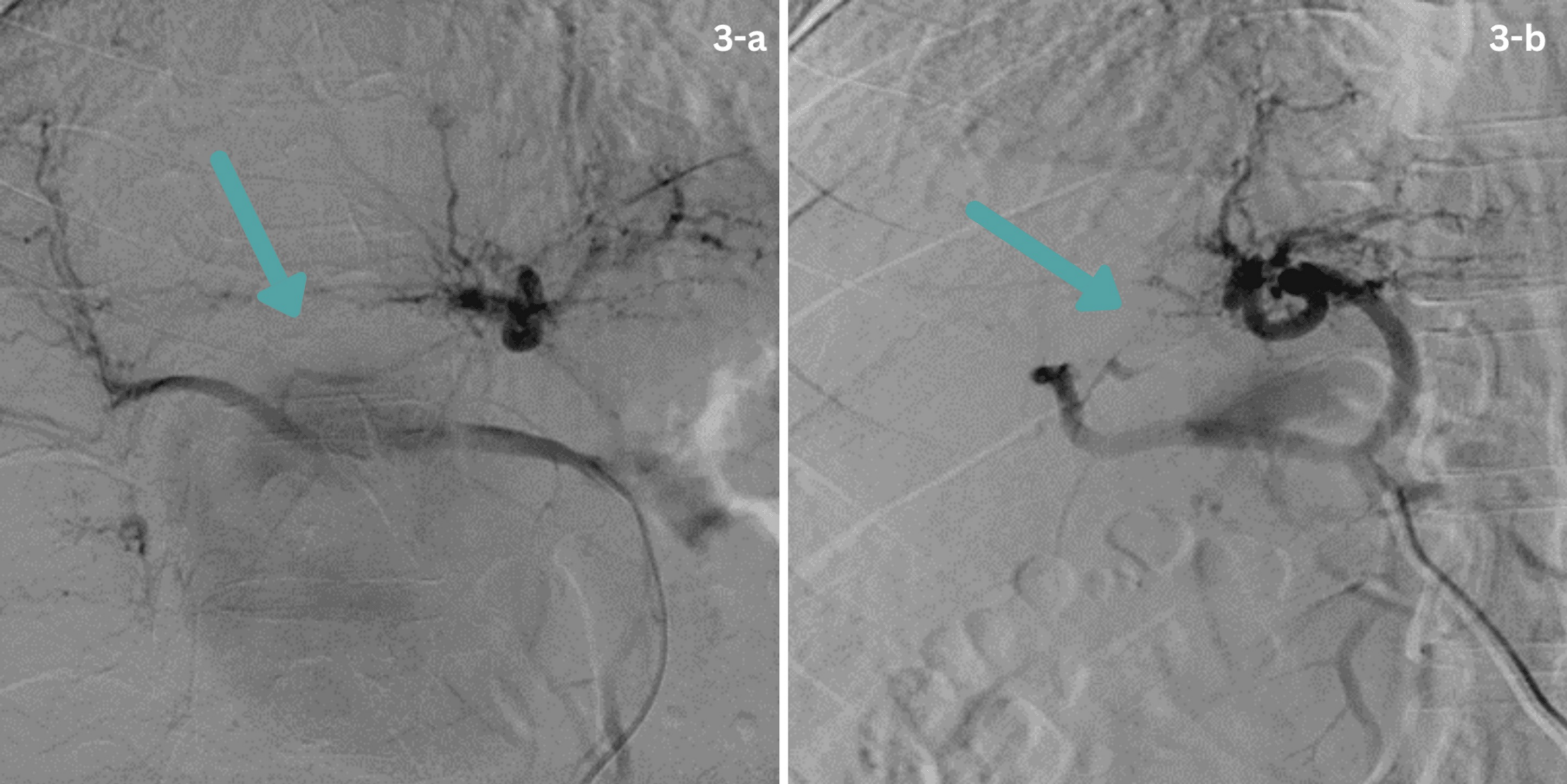
Digital substraction angiography (DSA) showing the right hepatic artery pseudoaneurysm and post-stenting exclusion. (a) DSA of the right hepatic artery demonstrating contrast opacification of a large pseudoaneurysm; a guidewire is positioned in preparation for endovascular treatment. (b) Post-stenting DSA image showing successful exclusion of the right hepatic artery pseudoaneurysm with restored arterial flow.

These endoscopic findings prompted an urgent CT angiogram, which showed a large HAP measuring 8.6 x 8 x 9 cm in the subhepatic region. A multidisciplinary team meeting recommended interventional radiology (IR)-guided stent placement across the hepatic artery aneurysm. The patient was shifted to the IR suite. Vascular access was achieved via the right femoral artery, and the catheter was advanced up to the common hepatic artery origin. Digital subtraction angiography of the hepatic arterial system revealed a large pseudoaneurysm. A guidewire was placed across the aneurysm, followed by successful placement of a stent across the pseudoaneurysm in the right hepatic artery (Figures 3a, 3b).

Post-procedure course was uneventful, and the patient was discharged after 48 hours of observation. No further episodes of bleeding were reported at a three-month follow-up in the outpatient department.

## Discussion

Laparoscopic cholecystectomy is a routinely performed surgical intervention with associated complications. Major complications include bleeding from the adjacent tissue (2.83%), cystic artery (0.67%), abdominal wall port (1.21%), and, rarely, ligaments of the liver (0.54%). Other complications comprise iatrogenic perforations of the gallbladder (5.27%), injuries to the common bile duct (0.13%), and spilled gallstones (2.02%) [9]. Pseudoaneurysm of the hepatic and/or cystic artery represents a rare complication following laparoscopic cholecystectomy. A pseudoaneurysm is formed when a damaged artery bleeds into the surrounding tissues and forms a cavity outside the vessel wall. It can be distinguished from a hematoma because it continues to communicate with the arterial lumen, forming a high-pressure cavity with a risk of rupture.

The spectrum of presentation for pseudoaneurysm ranges from vague abdominal pain, anemia, and jaundice to life-threatening hematemesis or melena. Hemobilia has been identified as the most common (85%) channel for GI bleeding resulting from HAP. The hepatic artery has been reported as the most frequent culprit in hemobilia (88.1%), followed by the cystic artery (7.9%) and a combination of both (4.0%), with an overall mortality rate of 2.0% [10-12]. In this case report, the aneurysm eroded through the duodenal wall in the form of a large ulcer with intermittent bleeding.

Imaging modalities such as ultrasound and contrast-enhanced CT or MRI play an important role in detecting HAP. Subsequent angiography is the gold standard and should be performed as soon as possible when suspected by ultrasound and enhanced CT or MRI [13]. It not only aids in diagnosis confirmation but also serves as a therapeutic modality. Literature has shown that endovascular therapy remains the primary management strategy, followed by surgical intervention. Pre-procedure imaging and angiography play a vital role in guiding the appropriate modality to manage the HAP. Various factors have been reported in the literature that deem endovascular therapy difficult to execute and include coexistent celiac axis stenosis, complex collateral circulation, challenging embolization of the pseudoaneurysm cavity, and the need for surgery to relieve the common bile duct obstruction [14]. In our case, the endovascular therapy by interventional radiologists successfully managed the HAP. Therefore, selecting endovascular therapy versus surgery depends on various factors, including the location of the pseudoaneurysm, operator experience, and multidisciplinary discussion is essential for selected patients, particularly for those with large pseudoaneurysms, complex collaterals, or special locations [13].

## Conclusions

HAP is a rare but serious complication following laparoscopic cholecystectomy. Patients with a history of a laparoscopic biliary surgery presenting with GI bleeding should be investigated for the possibility of a pseudoaneurysm. Endoscopic assessment and cross-sectional imaging, such as a CT scan or an MRI, are the preferred diagnostic modalities. Endovascular procedure is the most recommended treatment modality. Surgical management is reserved for patients in whom embolization remains unsuccessful and for those who are at risk of substantial hepatic infarction after embolization. Timely recognition through thorough history-taking, appropriate diagnostic evaluation, and prompt therapeutic intervention can lead to excellent long-term outcomes.
